# Ligand-lytic peptides for specific targeting of *Leishmania major* and *Trypanosoma cruzi* parasites

**DOI:** 10.3389/fcimb.2025.1595333

**Published:** 2025-05-30

**Authors:** Eva Iniguez, Felipe Rodriguez, Claudia Husseneder, Lane Foil, Rosa A. Maldonado

**Affiliations:** ^1^ Border Biomedical Research Center, Bioscience Research Building, Department of Biological Sciences, The University of Texas at El Paso, El Paso, TX, United States; ^2^ Department of Entomology, Louisiana State University Agricultural Center, Baton Rouge, LA, United States

**Keywords:** leishmaniasis, Chagas disease, lytic peptide, *Hecate*, antimicrobial peptide, chemotherapy

## Abstract

**Introduction:**

Leishmaniasis and Chagas disease are major human neglected diseases, affecting an estimate of 12 and 6 to 8 million people worldwide, respectively. Current treatments for both diseases are highly toxic for the vertebrate host and lack specificity for the parasites, highlighting the need for the discovery of new therapies against these diseases. In this study, we tested the use of the lytic peptide *Hecate* and a *Ligand-Hecate* construct that incorporates a ligand to bind the lytic peptide to protozoa membranes and screened them for protozoacidal activity.

**Methods:**

We first screened parasite survival of luciferase expressing *Leishmania major* promastigotes and *Trypanosoma cruzi* epimastigotes in the presence of *Hecate* or *Ligand-Hecate*, and after 12, 48 and 96 h by measuring the parasite luciferase activity. In addition, High-Content Imaging Assay was used to evaluate the proliferation of intracellular *L. major* amastigotes propagated inside murine macrophages after treatment with *Hecate* or *Ligand-Hecate*.

**Results:**

The lowest half maximal effective concentration observed after 48 h of incubation with *Hecate* and *Ligand-Hecate* was lower against *L. major* promastigotes than *T. cruzi* epimastigotes. *Ligand-Hecate* treatment significantly reduced infection rate of macrophages *L. major* amastigotes compared to the non-treated vehicle control; while treatment with *Hecate* was significant only at higher drug concentrations. Importantly, no significant cytotoxicity was observed when screened against intraperitoneal murine macrophages for either *Hecate* or *Ligand-Hecate* treatments.

**Discussion:**

Our results indicate that ligand-lytic peptide complexes are potential targets for therapeutic drugs that can selectively kill both extracellular and intracellular protozoa parasites stages with no significant toxicity to host cells.

## 1 Introduction

Chagas disease and leishmaniasis are major human vector-borne neglected tropical diseases caused by the related hemoflagellate parasites *Trypanosoma cruzi* and *Leishmania* spp. In 2019, approximately 6 to 8 million people were estimated to be infected with *T. cruzi*, and an additional 70 million were estimated to be at risk of infection (Lidani et al., 2019). The World Health Organization considers leishmaniasis to be an emerging and uncontrolled disease complex (Anon, 2024), with an estimated 1 million new cases annually in 2012 while 12 million people were considered to be infected (Alvar et al., 2012). Since then, the number of cases of cutaneous leishmaniasis, which is the most common and transient form of the disease, peaked in 2019 with 280,679 reported cases (Anon, 2024).

Leishmaniasis is transmitted by sand flies, which egest the infectious metacyclic stage of the parasite into the vertebrate host upon taking a blood meal (Serafim et al., 2021). The disease has a broad clinical spectrum, causing fatal visceral, cutaneous, and mucocutaneous leishmaniasis. Cutaneous leishmaniasis is the most common clinical manifestation that is caused by *L. major* in the Old World and may lead to disfiguring ulcerative lesion that occurs at the site of infection in humans (Desjeux, 2004). The life cycle of *Leishmania* parasites includes the extracellular promastigote stages within the sand fly vector and an intracellular amastigote form which inhabits the macrophages of the vertebrate host (Mann et al., 2021; Serafim et al., 2021).

The life cycle of *T. cruzi*, the vector for Chagas disease, starts when the trypomastigote (blood form) is ingested by blood-sucking triatomine bugs during a blood meal. Then, the parasites reproduce and transition from epimastigotes to the infectious metacyclic trypomastigotes in the insects’ gut. Upon taking a blood meal, the infectious metacyclic trypomastigotes are transmitted to the vertebrate host from infectious insect feces by either rubbing them at the bite site of the triatomine bug or by ingestion of the infected vector by the mammal. In the vertebrate host, the pathological human life stages of *T. cruzi* are the intracellular amastigotes (Cucunubá et al., 2024).

Current treatment options for leishmaniasis are limited to a small number of drugs, including arsenicals, amphotericin B, pentavalent antimonials, and anticancer alkyl-lysophospholipids that are expensive, toxic, and not fully effective (Croft et al., 1996; Mitropoulos et al., 2010). Nifurtimox and Benznidazole are the two most common drugs currently used to treat Chagas disease, however, these drugs are often associated with toxicity problems (e.g. nephrotoxicity and cardiotoxicity) and emergence of resistance (Berhe et al., 2024). In an effort to address the need for new and efficacious drugs, research studies have evaluated small peptide antimicrobial compounds for killing both trypomastigotes and amastigotes parasite stages (Vizioli and Salzet, 2002). Antimicrobial peptides (AMPs) are cationic proteins that are common components of the innate immune systems of many organisms, and studies have shown that host defense lytic peptides of both insects and vertebrates have cytotoxic activity toward *Leishmania* promastigotes and amastigotes (Perez-Cordero et al., 2011; Lynn et al., 2011). Lytic peptides are part of the nonspecific immune system of eukaryotes that destroy the membranes of microorganisms (Leuschner and Hansel, 2004; Guaní-Guerra et al., 2010) but are not likely to harm higher eukaryotes because they do not affect the electrically neutral cholesterol-containing cell membranes of higher eukaryotes (Javadpour et al., 1996; Bell, 2011).

McGwire and Kulkarni (2010) reviewed the interactions of AMPs found in aquatic animals, insects, plants and mammals with pathogenic *Leishmania* and *Trypanosoma* species. Multiple studies on insect AMPs used different forms of cecropin-like lytic peptides; the lytic peptide cecropin was first described from silkworm. Barr et al. (1995) used three peptides (DC-1, DC-2, and DC-2R) that were synthesized and had similar amphipathic and hydrophobic properties of cecropin B. The authors showed that all three peptides were effective in killing 100% of *T. cruzi* trypomastigotes *in vitro* at a concentration of 10 μM. In addition, a significant reduction in amastigote numbers in infected Vero cells was observed for all three peptides at 2.5 μM, with no toxicity at that concentration. The peptide DC-1 (*Hecate*) was tested intravenously for toxicity in mice with no observed effects at 50 micrograms daily for 10 days, and a significant reduction in *T. cruzi* parasitemia in infected mice was observed for mice treated 5 times over 10 days with 25 micrograms IV. DC-2 was effective *in vitro* at less than 1 μM and ranked at 4+ on a 1-4+ scale, placing it among the most potent AMPs in the insect category along with attacin and cecropin-melittin hybrids (McGwire and Kulkarni, 2010).

Lytic peptide action can be targeted to specific cell types by the addition of a ligand. For example, Hansel et al. (2007) reported that lytic peptides conjugated with cancer cell membrane receptor ligands could be used to destroy breast cancer cells, while lytic peptides alone or conjugated with non-specific peptides were not effective. Lytic peptides also have been conjugated with human hormones that bind to receptors on tumor cells for targeted destruction of prostate and testicular cancer cells (Leuschner and Hansel, 2004). Phage display technique was used to identify peptides that attach to the vital lignocellulose-digesting flagellate protozoa (phylum Parabasalia) in the guts of workers of the Formosan subterranean termite, *Coptotermes formosanus* (Husseneder et al., 2010; Sethi et al., 2014). The authors selected and synthesized two of 19 candidates, attached the fluorophore EDANS, and confirmed binding to the protozoa *in vitro*. Then they fused the ligand that was associated with the variant surface glycoprotein of *T. brucei* with *Hecate (Ligand-Hecate*) and demonstrated that the fusion peptide killed the protozoa in the termite gut more effectively than *Hecate* alone *in vitro* and *in vivo*. In addition, the authors confirmed that ligands designed against Parabasalia protozoa also bind to at least 4 other phyla and are thus likely conserved enough to target trypanosomatid life stages (Husseneder et al., 2010; Sethi et al., 2014).

The purpose of this study is to determine if the concept of targeting extracellular life stages of *T. cruzi* and *L. major* and intracellular *L. major* using the ligands developed in prior studies (Husseneder et al., 2010; Sethi et al., 2014) with the synthetic lytic peptide *Hecate* (Barr et al., 1995) can be applied to provide safe and efficient parasite treatment. In the current study, we compared the antiparasitic activity and cytotoxicity of *Hecate* and *Ligand-Hecate* constructs against *L. major* promastigotes and *T. cruzi* epimastigotes and assessed the inhibition of *L. major* amastigote development in macrophages after treatment with these lytic peptides.

## 2 Materials and methods

### 2.1 Lytic peptide synthesis

Two fluorescently labeled protozoa-specific ligands (*Ligand* 1: ALNLTLH, *Ligand* 2: LPSLPAN, Sethi et al., 2014) were synthesized at the Louisiana State University AgCenter Biotechnology Laboratory by coupling them to the fluorophore *EDANS* (5-((2-Aminoethyl) amino) naphthalene-1-sulfonic acid) via solid state peptide synthesis using NovaTag resin (EMD Biosciences). In addition, fusion proteins consisting of the first ligand and the lytic peptide Hecate (ALNLTLH-FALALKALKKALKKLKKALKKAL, referred to as *Ligand-Hecate*) were synthesized along with standalone *Hecate* (FALALKALKKALKKLKKALKKAL).

### 2.2 Trypanosomatid cultures

Epimastigote forms of *T. cruzi* DM28C strain expressing luciferase (*Tc-*luc) were grown in liver infusion-tryptose medium (Iniguez et al., 2013; Camargo, 1964). Promastigote forms of *L. major* strain Friedlin clone V1 expressing luciferase (*Lm*-luc) were grown in M199 medium supplemented with hemin, 10% inactivated fetal bovine serum (iFBS), 1% of 10,000 units/ml penicillin and 10 mg/ml streptomycin (Thalhofer et al., 2010).

### 2.3 Culture of intraperitoneal murine macrophages

Intraperitoneal murine macrophages (IPM) were obtained as previously described (Capul et al., 2007) and cultured in Dulbecco’s Modified Eagle’s Medium (DMEM), supplemented with 10% iFBS, along with 1% of 10,000 units/ml penicillin and 10 mg/ml streptomycin. The procedure was performed following the NIH guidance and animal protocol approved by UTEP’s Institutional Animal Care and Use Committee (IACUC).

### 2.4 Test for binding of *Ligand-EDANS* to *Leishmania major* promastigotes

The *L. major* promastigotes were pelleted by centrifugation and washed 3 times with 1X PBS. Cells were incubated for 1 h at 28°C with Alexa Fluor 488 Phalloidin (Thermo Fisher Scientific) at 1:500 dilution and the two ligands conjugated to *EDANS* were then added at a final concentration of 50 μM. After incubation, cells were washed and fixed with 4% paraformaldehyde for 15 min at room temperature. Then all samples were added to a Nunc Lab-Tek II Chamber Slide™ (Thermo Fisher Scientific) and incubated at 37°C for 2 h. *Ligand-EDANS* binding to *L. major* promastigotes was assessed by LSM 700 Zeiss confocal microscope. *Ligand*-*EDANS* at excitation of 341 and emission of 471 nm, and Alexa Fluor 488 Phalloidin at excitation of 495 and emission of 518 nm. The control was treated with Alexa Fluor 488 Phalloidin only.

### 2.5 Luciferase assay to test lytic peptide activity against *Leishmania major and Trypanosoma cruzi*

The anti-parasitic activity of *Hecate* and *Ligand-Hecate* was determined using the luciferase expressing *L. major* (*Lm*-luc) and *T. cruzi* (*Tc*-luc) strains described above. Briefly, *L. major* promastigotes and *T. cruzi* epimastigotes (10^6^/well) were tested in a two-fold serial dilution of *Hecate* and *Ligand-Hecate* ranging from 0.23, 0.46, 0.93, 1.87, 3.75, 7.5, 15, to 30 μM. Parasite survival was measured by luciferase activity with the substrate 5´-fluoroluciferin (ONE-Glo Luciferase Assay System, Promega) after 12, 48 and 96 h incubation at 28°C using a luminometer (Luminoskan, Thermo). The assay was performed in triplicate, and the half maximal effective concentration (EC_50_) was determined.

### 2.6 Assessment of cytoxicity of lytic peptides against murine macrophages with Alamar Blue assay

Alamar Blue is a fluorometric assay used to detect cell viability in a large range of cell types (Lara et al., 2010). Intraperitoneal murine macrophages (10^6^/well) were plated in a 96 well microplate, followed by the addition of the *Hecate* and *Ligand-Hecate* at concentrations of 0.23, 0.46, 0.93, 1.87, 3.75, 7.5, 15, and 30 μM. After 48 h of incubation at 37°C, 5% CO_2_, the toxicity to murine macrophages was determined by Alamar Blue (Invitrogen), as previously described (Lara et al., 2010). The assay was performed in triplicates from which the inhibitory concentrations (IC_50_) were determined. Therapeutic indices for the lytic peptides against *L. major* and *T. cruzi* were calculated from the ratio of IC_50_ of IPM to EC_50_ (see above) after lytic peptide exposure for 48h.

### 2.7 Proliferation of *L. major* measured by high-content imaging assay

Intraperitoneal murine macrophages (IPM) were obtained as previously described (Capul et al., 2007) and seeded at a density of 10^6^/well in a 96-well microplate. After 2 h of incubation, IPM were infected with metacyclic promastigotes of *Lm*-luc. The infection of macrophage cells was performed for 24 h, at a ratio of 10 to 1 parasites per macrophage. After 24 h, the infected cells were incubated with 0.41, 0.81, 1.62, 3.21, 7.5 and 15 µM *Hecate* and *Ligand-Hecate* for 48 h. The cells were fixed with 4% paraformaldehyde and stained with Alexa Fluor 488 Phalloidin (Thermo Fisher Scientific) and DAPI (Thermo Fisher Scientific). The percentage of infected cells with one or more amastigotes per cell was determined by high-content imaging assay using an IN Cell 2000 analyzer bioimaging system (GE Healthcare). A vehicle control (1% water) and a treatment control (5 μM Amphotericin B) were included as negative and positive control.

### 2.8 Statistics

Statistical tests were conducted with IBM SPSS Statistics version 24. A Univariate General Linear Model was employed to test for effects of the factors “Concentration” (0.23-30 μM of lytic peptide), “Incubation time” (12, 48, and 96 h), and Lytic peptide” (*Hecate* vs. *Ligand-Hecate*) on the viability of the parasites (*L. major* and *T. cruzi*). Bonferroni *post-hoc* multiple comparison tests were used for “Concentration” and “Incubation time”. Effects were considered significant for P≤0.05 and marginal for P>0.05<0.10. Effect size was measured using Partial Eta Square. One-sample t-tests were used to assess cytoxicity of lytic peptides against IPM compared to the 100% survival rate of the untreated vehicle control (1% water). Two-tailed paired-samples t-tests were employed for comparison of reduction in infection rates of macrophages after treatment with *Hecate* and *Ligand-Hecate* and to compare treatment efficiency across a range of concentrations to untreated infected macrophages and infected macrophages treated with 5 μM Amphotericin B.

## 3 Results

### 3.1 Ligand binding to *Leishmania major* promastigotes *in vitro*

**Figure 1:**
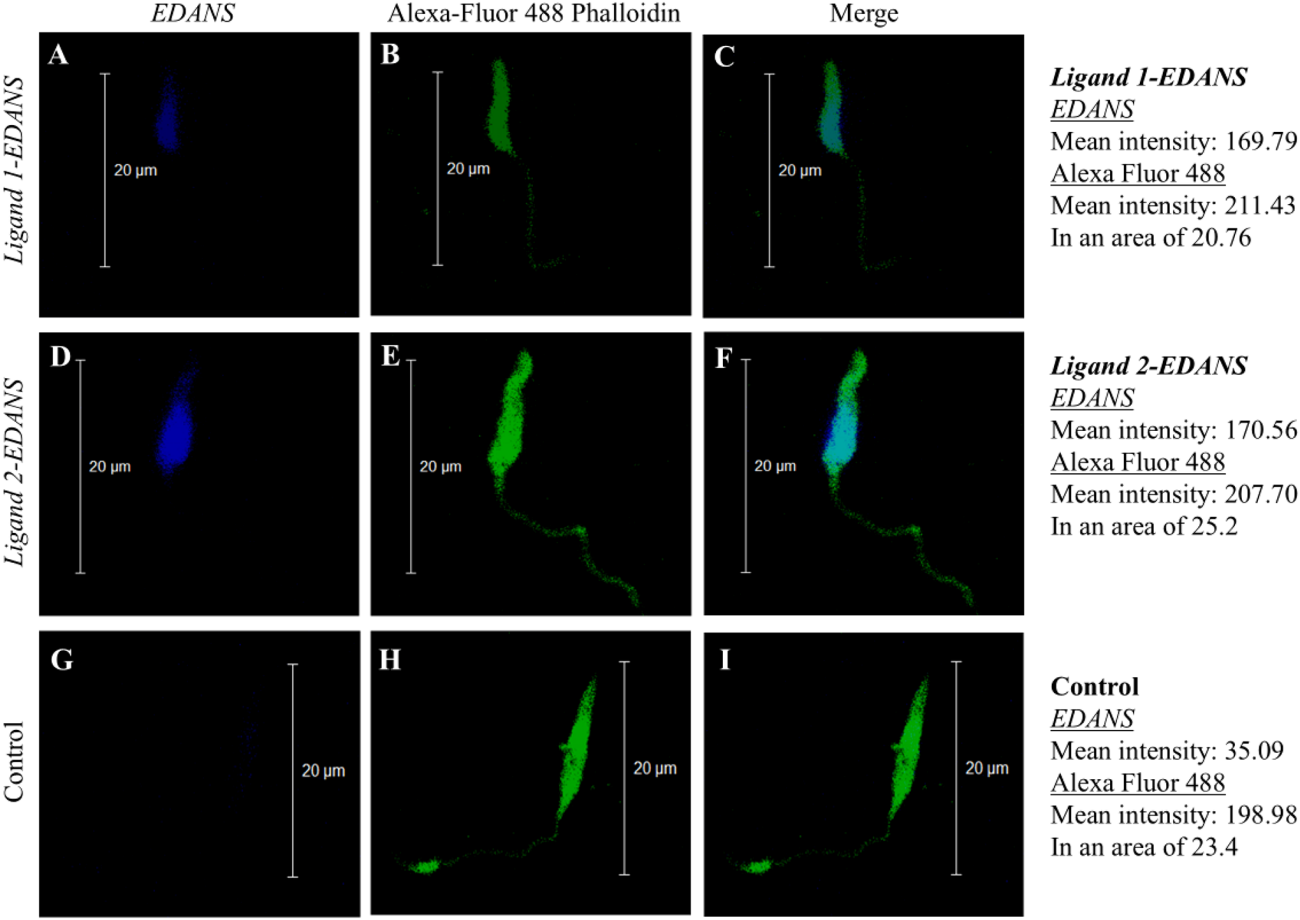
Fluorescence shows binding of *Ligand 1-EDANS* **(A–C)** and *Ligand 2-EDANS* **(D–F)** to *L. major* promastigotes. Binding of *Ligands* was observed by blue fluorescence in the *EDANS* only (*EDANS*) and the merged *EDANS* and Alexa Fluor 488 Phalloidin (Merge) image pannels. Control: Promastigotes with no *ligand-EDANS* **(G–I)**.

Promastigotes of *L. major* treated with *Ligand* 1*-EDANS* (**Figures 1A–C**) and *Ligand* 2-*EDANS* (**Figures 1D–F**) exerted a higher *EDANS* fluorescence mean intensity of 169.79 and 170.56, respectively, in comparison to the negative control (**Figures 1G–I**) with only 35.09. Therefore, we established that both ligands bound to *L. major* promastigotes. Comparison to Alexa Fluor 488 actin stains suggests that both ligands attached across the cell membrane. Since both ligands showed the same binding patterns in terms of location and intensity of fluorescence, only *Ligand* 1 was chosen for conjugation with *Hecate* and testing for parasiticidal activity.

### 3.2 Anti-parasitic activity and cytotoxicity of *Hecate* and *Ligand-Hecate*

First, the anti-parasitic activity of *Hecate* alone and *Ligand-Hecate* against *T. cruzi-Luc* DM28c epimastigotes and *L. major-Luc* promastigotes was measured at a 2-fold serial dilution ranging from 0.23 to 30 to 0.23 μM. Additionally, the possible toxicity of the *Hecate* and *Ligand-Hecate* toward IPM was assessed via cytotoxicity assays.

Factors “Concentration” (0.23-30 µM), “Incubation time” (12, 48, 96 h) and “Organism” (*L. major* vs *T. cruzi*) showed significant effects on the survival of parasites with significant interaction among the three factors (P<0.0001, Univariate GLM). Effect size (partial Eta squared) was highest for “Concentration” accounting for 98% of the variability, followed by “Incubation time (62%) and “Organism” (46%). No overall effect of “Lytic peptide” (*Hecate* vs *Ligand-Hecate*) on parasite survival was detected (P=0.16).

**Figure 2:**
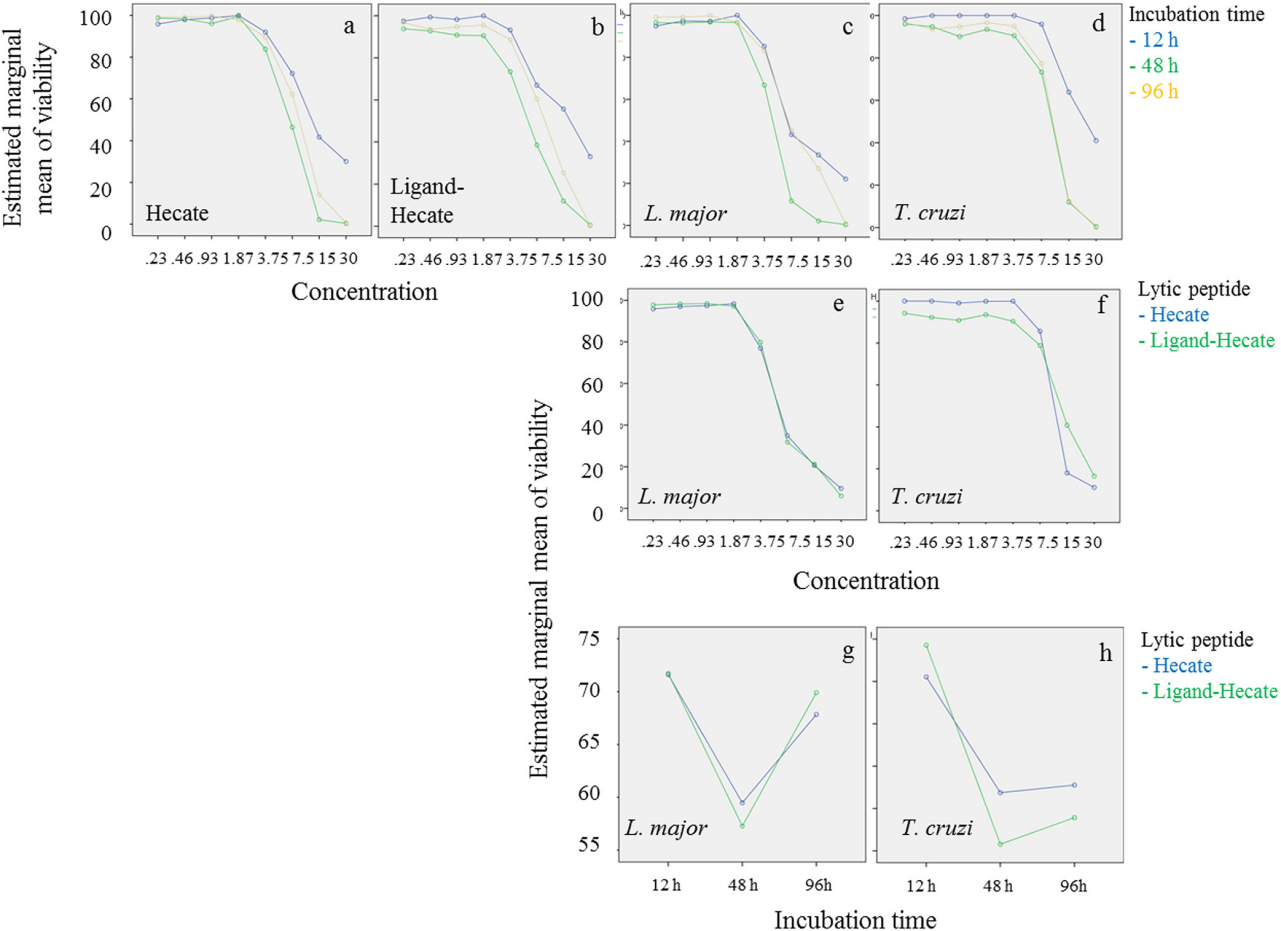
Parasite viability at different concentrations of lytic peptides and incubation times. Profile plots (Univariate General Linear Model, SPSS) show that both *Hecate* and *Ligand-Hecate* work most efficiently at 48 h incubation time against both parasite species **(A–D, G, H)**. *Ligand-Hecate* was more efficient in lower concentrations at 48 h incubation time than *Hecate* but not at the highest concentration **(A, B)**. *Leishmania major* was more sensitive to lytic peptide action than *T. cruzi* **(C–F)**. *Hecate* and *Ligand-Hecate* were equally efficient against *L. major* **(E, G)** but *Ligand-Hecate* showed superior performance against *T. cruzi* at lower concentrations **(F)** and longer incubation times **(H)**.

**Figure 3:**
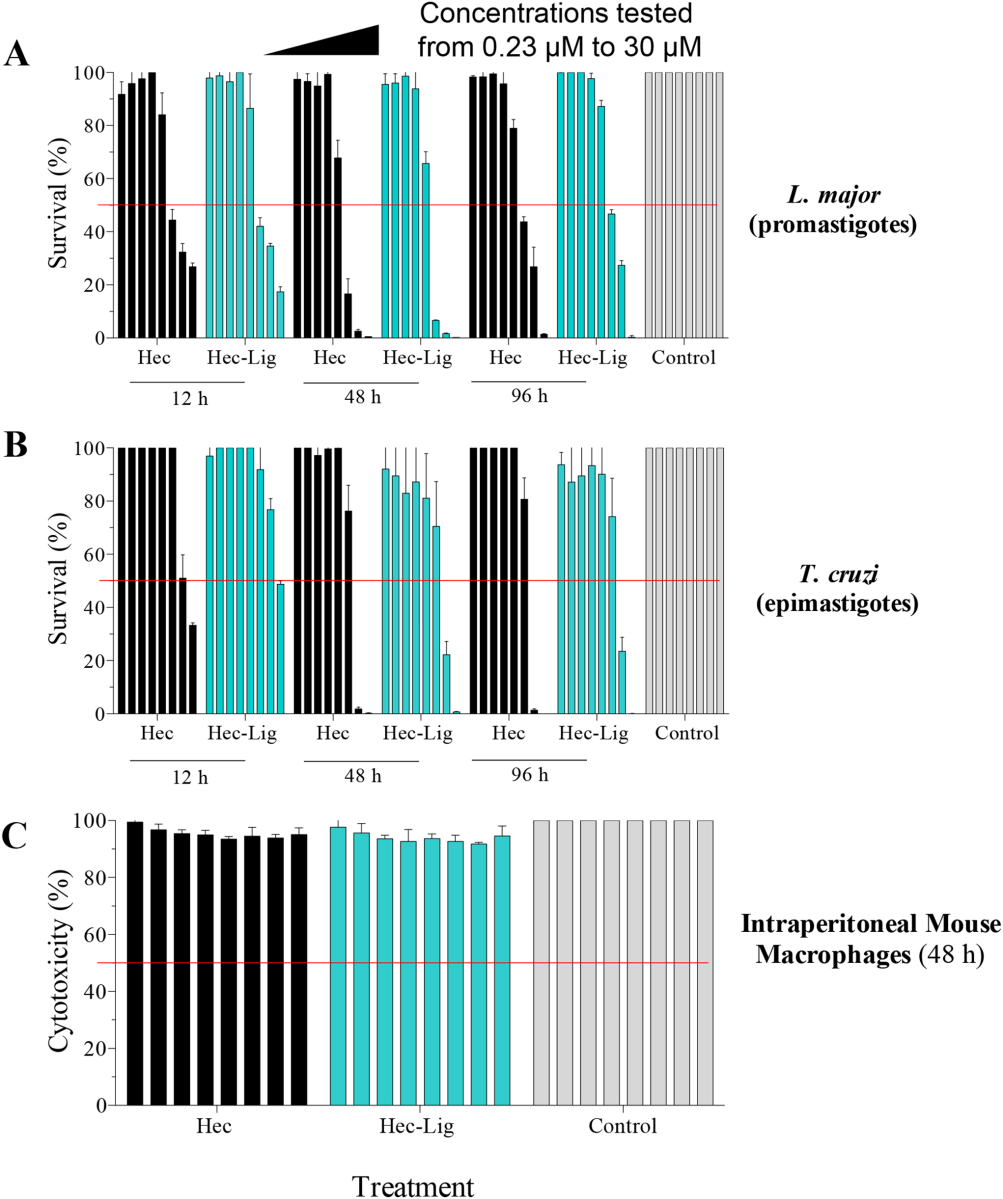
*In vitro* antiparasitic activity and cytotoxicity of *Hecate* and *Ligand-Hecate*. **(A)** Viability assay of *L. major* promastigotes incubated with *Hecate* (Hec) and *Ligand-Hecate* (Lig-Hec) at different time points (12, 48 and 96 h). Control treated with 1% water. **(B)** Viability assay of *T. cruzi* epimastigotes incubated with *Hecate* (Hec) and *Ligand-Hecate* (Lig-Hec) at different time points (12, 48 and 96 h). Control treated with 1% water. **(C)** Cytotoxicity assay in intraperitoneal murine macrophages incubated with *Hecate* (Hec) and *Ligand-Hecate* (Lig-Hec) for 48 (h) Control treated with 1% water. Concentrations tested at a range of 0.23, 0.46, 0.93, 1.87, 3.75, 7.5, 15, and 30 μM. Graphs show the mean +/-SD of the triplicate.

Lytic peptide treatment caused 50% reduction in survival at lower concentrations of both lytic peptides (between 3.8 and 7.5 µM) in *L. major* than *T. cruzi* (between 7.5 and 30 μM) across all incubation times (**Figures 2**, **3**). Viability of both parasite species was lowest at 48 h when treated with *Hecate* and *Ligand-Hecate* (**Figures 2A–D, G, H**). *Ligand-Hecate* was more efficient in lower concentrations at 48 h incubation time than *Hecate* but not at the highest concentration (**Figures 2A, B**). *Hecate* and *Ligand-Hecate* were equally efficient against *L. major* (**Figures 2E, G**) but *Ligand-Hecate* showed superior performance against *T. cruzi* at lower concentrations (**Figure 2F**) and longer incubation times (**Figure 2H**).

Similar to viability, incubation time for 48 h showed the most effect in terms of the lowest EC_50_ of *Hecate* and *Ligand-Hecate* against both *T. cruzi* epimastigotes (EC_50_: 8.8 and 10.4 μM) and *L. major* promastigotes (4.9 and 4.4 μM) (**Figure 3**; **Supplementary Table 1**). The therapeutic indices of both lytic peptides at 48h incubation time were higher against *L. major* (*Hecate*: >6.1, *Ligand-Hecate*: >6.8) than *T. cruzi* (*Hecate*: >3.7, *Ligand-Hecate*: >2.9).

No significant cytotoxicity of *Hecate* and *Ligand-Hecate* was observed against IPM at 48 h incubation in the lower concentration range (0.23 and 0.46 μM) and at the highest concentration (30 μM; 2-tailed one-sample t-test) as well as for 1.28 μM of *Ligand-Hecate* and 7.5 μM of *Hecate*. Although significant in some of the mid-range concentrations (0.9-15 μM), the cytotoxicity produced by both lytic peptides was small with a survival rate above 90%.

### 3.3 High-content imaging assay to study the proliferation of intracellular *L. major* amastigotes propagated inside murine macrophages after treatment with lytic peptides

**Figure 4:**
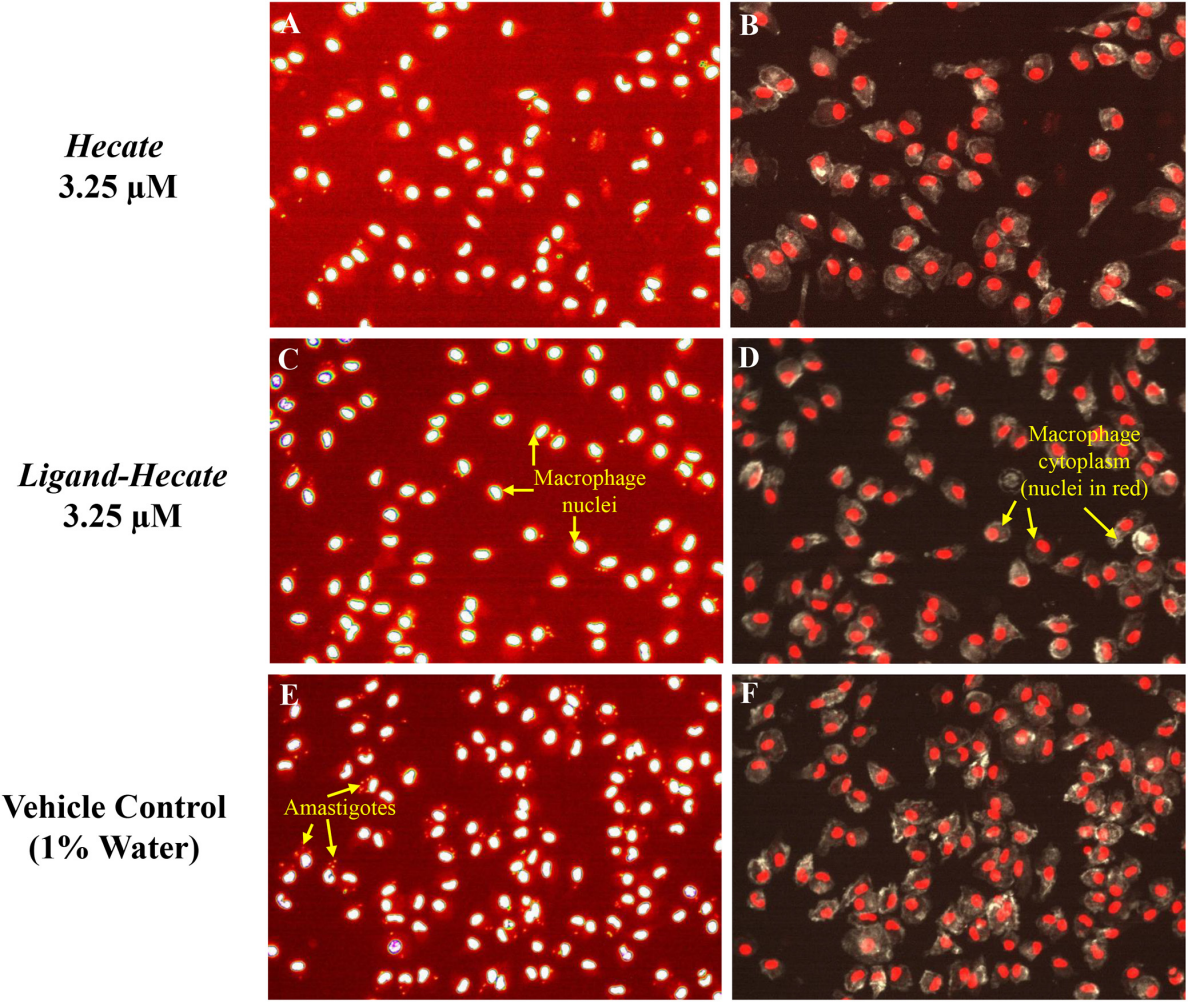
*Hecate* and *Ligand-Hecate* antiparasitic activity in infected macrophages Representative pictures of the effect of *Hecate* and *Ligand-Hecate* on the proliferation of *L. major* amastigotes propagated in mice intraperitoneal macrophages stained with DAPI **(A, C, E)**. At 3.25 μM both lytic peptides reduced the infection rate approximately by half. Representative segmentation images of macrophages stained with DAPI for the nucleus and *L. major* amastigotes and Alexa Fluor 488 Phalloidin for the cytoplasm **(B, D, F)**. Intact cytoplasm shows lack of toxicity of the tested lytic peptides against macrophages. Arrows point to amastigotes (size typically 2-4 μm), nuclei (ca. 5 μm) and macrophages (typically 10-20 μm in culture).

**Figure 5:**
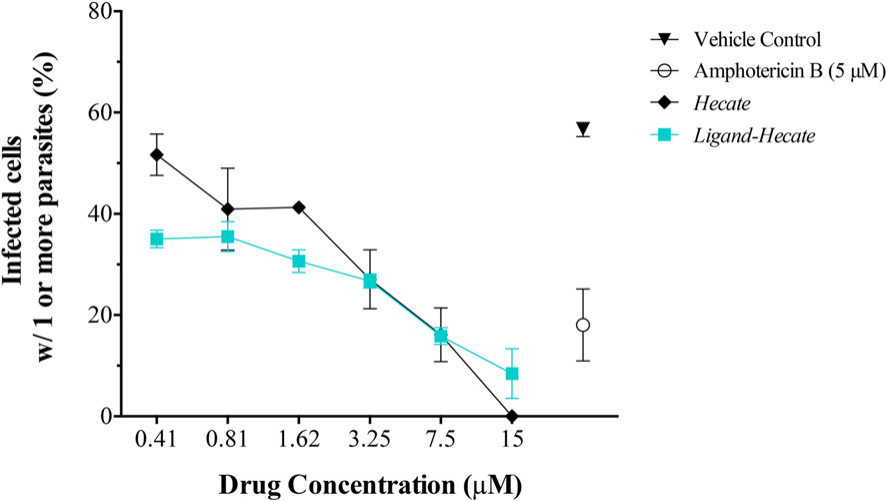
Effect of *Hecate* and *Ligand-Hecate* on the proliferation of *L. major* amastigotes propagated in mice intraperitoneal macrophages. The percentage of infected macrophages with one or more amastigotes was determined after treatment with different concentrations of *Hecate* and *Ligand-Hecate* and compared to the infection rate after treatment with 5 μM Amphotericin B (positive control) and the untreated vehicle control (1% water). Graphs show the mean +/-SD of the triplicate.

Since *Hecate* and *Ligand-Hecate* killed *L. major* promastigotes with low toxicity against murine macrophages, the therapeutically more relevant infectious intracellular amastigotes form of *L. major* was evaluated by high-content imaging assay to study the proliferation of intracellular amastigotes propagated inside intraperitoneal macrophages (IPM). Treatment with both *Hecate* and *Ligand-Hecate* resulted in inhibition of the proliferation of intracellular amastigotes (**Figures 4**, **5**).

As visualized in the representative segmentation pictures in **Figure 4**, the number of parasites was reduced by about 50% in IPM treated with 3.25 μM *Hecate* (**Figures 4A, B**) and *Ligand-Hecate* (**Figures 4C, D**) in comparison to the vehicle control (1% water) (**Figures 4E, F**). No significant cytotoxicity for IPM was observed for either *Hecate* and *Ligand-Hecate* (**Figures 4B, D**) as the morphology of the IPM is intact after the treatment with the compounds.

*Ligand-Hecate* treatment significantly (P<0.003, df=3) reduced infection rate of macrophages compared to the non-treated vehicle control (1% water) at all concentrations tested (0.41-15 μM, **Supplementary Table 2**). *Hecate* treatment significantly reduced infection rates at concentrations ranging from 1.62 to 15 μM (P<0.039, df=2), but not for the concentrations below that threshold (P>0.16, df=2, 2-tailed pairwise t-test). The observed reduction of infection rates at the concentration of 0.81 μM and higher of both *Ligand*-*Hecate* and *Hecate* were not significantly different (all P>0.13, 2-tailed pairwise t-test) from the reduction in infection rate achieved by the anti-leishmaniasis drug Amphotericin B at a dose of 5 μM (**Supplementary Table 2**). These results underscore the anti-parasitic efficacy of both forms of the lytic peptide at low concentrations.

No difference was observed in the efficiency of *Hecate* with or without ligand at the higher concentration levels (3.25-15 μM). However, *Ligand-Hecate* reduced the infection rate significantly more than *Hecate* alone at the lowest concentration tested (0.41 μM, P=0.04, t= 4.626) and marginally more at 1.62 μM (P=0.06, t=3.791, df=2, 2-tailed paired-samples t-test). This suggests that a ligand that specifically binds lytic peptide to protozoa increases the efficiency as protozoacidal agent.

## 4 Discussion

As a first step towards efficient targeting of lytic peptides to trypanosomatids, we tested two ligands for their capacity to bind *L. major* promastigotes. These ligands were originally designed via phage display of linear random heptapeptides to identify termite protozoa-recognizing peptides (Husseneder et al., 2010; Sethi et al., 2014). Two out of 19 peptides were selected for ligand development and shown using fluorophore EDANS to bind with the symbiotic termite gut protozoa but not the gut wall *in vivo* (Husseneder et al., 2010). Initially, both ligands were tested and shown to attach across the cell membrane of *L. major* promastigotes. Since both ligands showed similar binding patterns, only *Ligand* 1 was chosen to synthesize fusion proteins with the lytic peptide *Hecate* (*Ligand-Hecate*) and testing for parasiticidal activity. *Ligand* 1 was selected because it showed homology to epitopes present on the variant surface glycoprotein of *Trypanosoma brucei.* Additionally, we had prior evidence that a *Ligand* 1-*Hecate* fusion protein successfully kills protozoan symbionts in termites and is evolutionary conserved enough to target membrane receptors across a variety of protozoan phyla (Husseneder et al., 2010; Sethi et al., 2014).

Herein, we showed that the *Hecate* and *Ligand-Hecate* lytic peptides were most effective in killing both *T. cruzi* epimastigotes and *L. major* promastigotes at 48 h. Importantly, both lytic peptides showed negligible cytotoxicity to intraperitoneal murine macrophages even at high concentrations across all treatment durations. Similarly, Barr et al. (1995) showed that *Hecate* had no toxicity effect on Vero cell cultures at 2.5 μM but did cause a significant reduction in the number of *T. cruzi* amastigotes in infected Vero cells when administered at a double exposure of 2.5 μM at 24 h and 48 h post-incubation. The therapeutic indices of *Hecate* and *Ligand-Hecate* were both higher against *L. major* promastigotes than *T. cruzi* epimastigotes.

The proliferation of intracellular *L. major* amastigotes propagated inside murine macrophages was significantly reduced after treatment with *Hecate* or *Ligand-Hecate* and both lytic peptides were equally successful in reducing the percentage of infected cells as 5 μM of the commercial trypanomiasis treatment Amphotericin B. However, the *Ligand-Hecate* treatment outperformed treatment with *Hecate* alone at lower concentrations. The addition of the ligand increased the efficiency of *Hecate* in inhibiting the proliferation of the *L. major* intracellular amastigotes at a 4X lower concentration (0.41 μM) when compared to Hecate alone (1.62 μM) and thus *Ligand-Hecate* ranks highest among AMPs previously tested (McGwire and Kulkarni, 2010). Similarly, Sethi et al. (2014) showed that *Ligand-Hecate* had increased efficiency over *Hecate* alone killing the symbiotic protozoa of termites faster than Hecate alone.

In the present study we only tested the lytic peptides against *T. cruzi in vitro*. However, Barr et al. (1995) did provide proof that *Hecate* reduced the development of parasitemia of *T. cruzi* in mice and was not toxic to mice. Furthermore, the authors showed that 100% of mice infected with *T. cruzi* died by day 14 post-infection, while mice treated with *Hecate* after infection showed no weight loss or mortality by this timepoint. Studies like the one of Barr et al. (1995) but adding ligands to *Hecate* would be the next step towards development of AMP therapy against trypanosomatids. Importantly, the performance of the *Ligand-Hecate* treatment tested in our study warrants future studies using available *in vivo* models for pathogenic species of *Trypanosoma* and *Leishmania*.

Our study indicates the therapeutic action of AMPs against both extracellular and intracellular protozoa parasites with no significant toxicity to host cells. However, the mechanisms of cytolysis by AMPs are not well understood. The AMPs are small cationic proteins of the innate immune system of organisms which contain high percentages of basic amino acids that form either *α*-helical of *β*-pleated sheets. The AMPs have been promoted as alternatives for conventional antiprotozoal drugs because of low toxicity and less likely occurrence of resistance (Giovati et al., 2018). The amphipathicity of AMPs enables them to interact with negatively charged membranes and destabilize surface-membranes through a variety of mechanisms (McGwire and Kulkarni, 2010). Mutwiri et al. (2000) studied the potential mechanisms for membrane disruption of *Trichomonas* spp. caused by treatment with *Hecate*. The authors used scanning electron microscopy to demonstrate the extensive damage to plasma membranes of the trichomonads caused by 10 ppm *D-Hecate* in just 10 min. Mutwiri et al. (2000) concluded that time dependent accumulation of high concentrations of peptides was more likely to contribute to membrane disruption and cell disintegration rather than changes osmotic pressure.

The need for and approaches to developing conjugates to increase specificity and efficacy of AMPs varies by targeted cell membranes, hosts, and desired outcomes. Studies have shown that lytic peptides conjugated with cancer cell membrane receptor ligands or human hormones destroy tumor cells while lytic peptides alone or conjugated with non-specific peptides were not effective (Leuschner and Hansel, 2004; Hansel et al., 2007). Similarly, the ligand designed via phage display to attach to termite protozoa, protected non-targets like bacteria and termite gut tissue from lytic peptide action when fused to *Hecate* (Husseneder et al., 2010; Sethi et al., 2014), while efficiently killing cellulose digesting symbiotic protozoa of Formosan subterranean termites *in vitro* and *in vivo*. The same *Ligand-Hecate* construct was used in the present study to target *L. major* and *T. cruzi*, indicating conserved membrane receptors across distant phyla of protozoa. While we did not compare specificity of *Hecate* vs. *Ligand-Hecate* in this study, Sethi et al.'s (2014) results did provide evidence for the protective effect of adding a ligand to the lytic peptide to increase specificity and reduce off target effects against bacteria and insect tissue. Many studies showed that AMPs can be used *in vitro* to kill protozoa, but development of ligands can provide the flexibility and specificity to address *in vivo* success to kill protozoa with small peptide chemistries. The use of AMPs for vector borne pathogens is not limited to developing safe and effective treatments for trypomastigote parasites affecting humans. Moreira et al. (2007) showed that the *in vitro* growth of intracellular forms of *Plasmodium falciparum*, the causative agent of Malaria, was inhibited when treated with the spider AMP gomesin. Furthermore, *Anopheles stephensi* mosquitoes infected with *P. falciparum* or *P. berghei* that fed on mice treated with gomesin failed to produce the usual or expected number of oocysts (Moreira et al., 2007). Therefore, the area-wide use of AMPs for curative therapy of malaria in humans also could lower the infection rates of mosquitoes in communities.

The use of AMPs for control of transmission of *T. cruzi* in human domiciles has been promoted. Beard et al. (2002) conducted studies using a transgenic bacterial symbiont (*Rhodococcus rhodnii*) of *Rhodnius prolixus* to express Cecropin A resulting in the death of *T. cruzi* trypomastigotes in the gut of the vector. The genetically modified symbionts were delivered in a synthetic paste to simulate feces of *R. prolixus*, which is a source of symbiotic bacteria for first instar bugs via coprophagy. Sethi et al. (2014) employed a similar paratransgenesis approach for termite control by targeting and killing cellulose-digesting gut protozoa of Formosan subterranean termites. The authors genetically engineered yeast to express *Ligand-Hecate* and incorporated freeze-dried transgenic yeast into a cellulose bait. Termite foragers ingested the yeast, transferred it among colony member workers via coprophagy and trophallaxis, and the loss of protozoa killed the termite colony within weeks. The use of a transgenic yeast rather than a bacterium likely would add environmental stability for baits and facilitate scaled-up drug production for targeting reservoir vertebrates or invertebrates aiming to break transmission cycles.

In conclusion, most of the drugs currently used for treatment of Chagas disease and leishmaniasis have high vertebrate toxicity and low specificity for the parasites. The ligand-based drug design approach has been reported for improving or identifying new chemotypes (Berhe et al., 2024). Our study was an *in vitro* demonstration of efficacy and safety of *Ligand-Hecate* for killing intracellular and extracellular life stages of *Leishmania* and extracellular stages of *Trypanosoma* parasites. The addition of the ligand increased the lytic peptide action at lower concentrations. We also showed that lytic peptide (*Hecate*) treatment alone is safe but gains efficiency for curative *L. major* amastigote therapy with a ligand-based design. The efficacy of *Hecate* was increased by 4X (from 1.62 μM to 0.41 μM) by addition of a ligand known to bind with symbiotic protozoa of termites that showed homology to epitopes present on the variant surface glycoprotein of *Trypanosoma brucei* (Husseneder et al., 2010; Sethi et al., 2014). The efficacy at treatment levels less than 1μM rank highest among AMPs previously tested (McGwire and Kulkarni, 2010). Furthermore, this protozoacidal construct can be produced and delivered by transgenic yeast (Sethi et al., 2014). Therefore, the potential of using transgenic yeast or bacteria in the production of ligand-lytic peptides for therapy or even as delivery systems for humans, reservoir vertebrate hosts, and invertebrate hosts is an innovative approach to drug development.

## Data Availability

The original contributions presented in the study are included in the article/**Supplementary Material**. Further inquiries can be directed to the corresponding author.
