# Supplementary material for: Ligand-lytic peptides for specific targeting of Leishmania major and Trypanosoma cruzi parasites
Source: Front Cell Infect Microbiol. 2025 May 30;15:1595333. doi: 10.3389/fcimb.2025.1595333 (PMC12162945; doi:10.3389/fcimb.2025.1595333)
Supplement: Supplementary file 1 [file Table1.docx]

**Supplementary Table 1.** *In vitro* antiparasitic activity of *Hecate*, *Ligand-Hecate* against *Leishmania major* promastigotes and *Trypanosoma cruzi* epimastigotes, and cytotoxicity on intraperitoneal murine macrophages (IPM) at 48h incubation time. The therapeutic indices derived from the ratio of IC_50_ to EC_50_ were higher against *L. major* than *T. cruzi*.

| **Compound**  **(Incubation time)** | ***L. major***  **Promastigotes**  **EC_50_ (µM)** | ***T. cruzi***  **Epimastigotes**  **EC_50_ (µM)** | **Mammalian cells**  **IPM**  **IC_50_ (µM)** | **Therapeutic Index**  **(IC_50_/EC_50_)** |
| --- | --- | --- | --- | --- |
| **Hecate (12 h)** | **5.6** | **~ 14.2** | **n/a** | **n/a** |
| **Hecate (48 h)** | **4.9** | **8.8** | **>30** | **>6.1 (*L. major*)**  **>3.7 (*T. cruzi*)** |
| **Hecate (96 h)** | **6.4** | **8.6** | **n/a** | **n/a** |
| **Ligand-Hecate (12 h)** | **5.8** | **15.8** | **n/a** | **n/a** |
| **Ligand-Hecate (48 h)** | **4.4** | **10.4** | **>30** | **>6.8 (*L. major*)**  **> 2.9 (*T. cruzi*)** |
| **Ligand-Hecate (96 h)** | **6.8** | **11.1** | **n/a** | **n/a** |
