# Supplementary material for: Ligand-lytic peptides for specific targeting of Leishmania major and Trypanosoma cruzi parasites
Source: Front Cell Infect Microbiol. 2025 May 30;15:1595333. doi: 10.3389/fcimb.2025.1595333 (PMC12162945; doi:10.3389/fcimb.2025.1595333)
Supplement: Supplementary file 2 [file Table2.docx]

**Supplementary Table 2: Inhibition of *Leishmania major* amastigotes in macrophages by different Hecate and Ligand-Hecate concentrations**

Different letters across rows indicate significant difference in infection reduction compared between corresponding concentrations of Hecate and Ligand-Hecate and compared to infected macrophages (vehicle control) and infected macrophages after treatment with 5 μM amphotericin B (Amp B).
